# Long-term control of acromegaly after pituitary surgery in South-Eastern Norway

**DOI:** 10.1007/s00701-023-05772-7

**Published:** 2023-09-04

**Authors:** Camilla M. Falch, Anne K. Dupont, Nicoleta C. Olarescu, Markus Wiedmann, Daniel Dahlberg, Jens Bollerslev, Jon Berg-Johnsen, Ansgar Heck

**Affiliations:** 1 Section of Specialized Endocrinology, Department of Endocrinology, Oslo University Hospital, Postboks 4950 Nydalen, 0424 Oslo, Norway; 2 Institute of Clinical Medicine, Faculty of Medicine, University of Oslo, Postboks 1171 Blindern, 0318 Oslo, Norway; 3 Department of Neurosurgery, Oslo University Hospital, Postboks 4950 Nydalen, 0424 Oslo, Norway

**Keywords:** Growth hormone/somatotroph pituitary adenoma, Cure rate, Remission, Predictive factors, IGF-1

## Abstract

**Purpose:**

Sustained cure of acromegaly can only be achieved by surgery. Most growth hormone (GH) secreting pituitary adenomas are macroadenomas (≥ 10 mm) at diagnosis, with reported surgical cure rates of approximately 50%. Long-term data on disease control rates after surgery are limited. Our aim was to estimate short- and long-term rates of biochemical control after pituitary surgery in acromegaly and identify predictive factors.

**Methods:**

Patients operated for GH-secreting pituitary adenomas between 2005–2020 were included from the local pituitary registry (*n* = 178). Disease activity and treatment data were recorded at one-year (short-term) and five-year (long-term) postoperative follow-up. Biochemical control was defined as insulin-like growth factor 1 (IGF-1) ≤ 1.2 × upper limit of normal value. Multivariate regression models were used to identify factors potentially predicting biochemical control.

**Results:**

A total of 178 patients with acromegaly (median age at diagnosis 49 (IQR: 38–59) years, 46% women) were operated for a pituitary adenoma. Biochemical control was achieved by surgery in 53% at short-term and 41% at long-term follow-up, without additional treatment for acromegaly. Biochemical control rates by surgery were of same magnitude in paired samples (45% vs. 41%, *p* = 0.213) for short- and long-term follow-up, respectively. At short-term, 62% of patients with microadenomas and 51% with macroadenomas, achieved biochemical control. At long-term, the biochemical control rate was 58% for microadenomas and 37% for macroadenomas (*p* = 0.058). With adjunctive treatment, 82% achieved biochemical control at long-term. Baseline IGF-1 levels significantly predicted biochemical control by surgery at short-term (OR: 0.98 (95% CI: 0.96–0.99), *p* = 0.011), but not at long-term (OR: 0.76 (95% CI: 0.57–1.00), p = 0.053).

**Conclusion:**

In unselected patients with acromegaly, the long-term biochemical control rate remains modest. Our findings indicate a need to identify patients at an earlier stage and improve therapeutic methods and surgical outcomes.

**Supplementary Information:**

The online version contains supplementary material available at 10.1007/s00701-023-05772-7.

## Introduction

Acromegaly is usually caused by a pituitary adenoma with persistent growth hormone (GH) hypersecretion and consecutively elevated insulin-like growth factor 1 (IGF-1) levels [16]. Management of patients is recommended to be centralized to tertiary referral centers with a multidisciplinary and individualized approach, often requiring multimodal treatment including surgery, medical and/or radiation therapy [6]. Surgery, usually by a transsphenoidal approach, is the only curative treatment. However, there is considerable variation in primary cure rates from 51–87% for microadenomas (< 10 mm) and 35–67% for macroadenomas (≥ 10 mm) [17, 21, 28, 29]. As most patients present with macroadenomas at diagnosis (around 70%), the overall cure rate is disappointingly low [6, 7]. Register-based studies of patients with acromegaly have described surgical cure rates of 34–40% [4, 21]. In a meta-analysis reporting pituitary surgery outcome within European centers, the overall remission rate in patients with acromegaly was 50% [35]. The reported cure rates vary considerably depending on study design and criteria for biochemical control that have been changing over time, making it challenging to compare study results [35]. Among earlier series describing surgical outcomes, only a few have long-term follow-up extending to more than three years [1, 7, 13, 19, 24]. Lower levels of preoperative GH and IGF-1, smaller and noninvasive tumors, and older age at diagnosis, are among factors that potentially predict surgical outcome and long-term biochemical control [11, 17, 30, 31, 33].

Our aim was to estimate short- and long-term rates of biochemical control after pituitary surgery in a large series of consecutively and newly diagnosed patients with acromegaly in South-Eastern Norway, and identify predictive factors for long-term biochemical control.

## Material and methods

### Patient characteristics and management

Patients diagnosed with acromegaly and treated surgically for a GH-secreting pituitary adenoma from 2005 to 2020 were eligible for inclusion. Patients were prospectively included in the pituitary registry at Oslo University Hospital (OUS); a tertiary referral center for patients with acromegaly in South-Eastern Norway, comprising approximately 3 million inhabitants. The patients were followed prospectively from diagnosis, until the five-year postoperative follow-up or end of study (November 30, 2022). For the present study, short- and long-term outcome was defined as the one-year and five-year postoperative follow-up, respectively. A multidisciplinary treatment approach according to the most recent recommendations [6, 14, 16, 18, 20] included surgical procedures, medical treatment (1^st^ generation somatostatin analogues (SSAs), pasireotide, dopamine agonists (DAs), GH receptor antagonists (GHRAs)) and/or radiotherapy. During the study period, different assays for GH and IGF-1 were used. For statistical analysis, random morning GH levels (μg/L) were used as described previously [23]. We present both absolute IGF-1 (nmol/L) and IGF-1/ULN (the ratio of measured IGF-1 values, divided by the age-specific upper limit of normal) values. Biochemical control was defined by IGF-1/ULN ≤ 1.2, as recommended [16]. Random GH levels < 1 µg/L were also evaluated as an additional criterion of biochemical control. As biochemical control status could not be assessed, follow-up with missing IGF-1/ULN values were excluded from the study. We investigated if biochemical control rates for micro- and macroadenomas at the five-year follow-up changed when biochemical control was defined as IGF-1 < 1 × ULN.

### Statistical analyses

Data are presented as median (interquartile range (IQR)) for continuous variables, and n (%) for categorical variables. Two by two tables were used to investigate biochemical control rates between groups. Wilcoxon rank-sum or Wilcoxon matched-pairs signed-rank test was used for comparison of medians, and chi-square or McNemar’s test was used for comparison of proportions, depending whether the data was paired or not. Multivariate analysis with backward stepwise regression was used to estimate association between biochemical control and age, sex, GH-, IGF-1 and IGF-1/ULN levels, tumor size (micro- vs. macroadenoma) and first treatment (surgery vs. medical pretreatment with SSAs), and data are expressed as odds ratios (OR) with 95% confidence intervals (95% CI). Two-tailed P-values < 0.05 were considered significant. Statistical analyses were performed by using STATA version 17.0.

## Results

### Patient characteristics and initial treatment

**Fig. 1:**
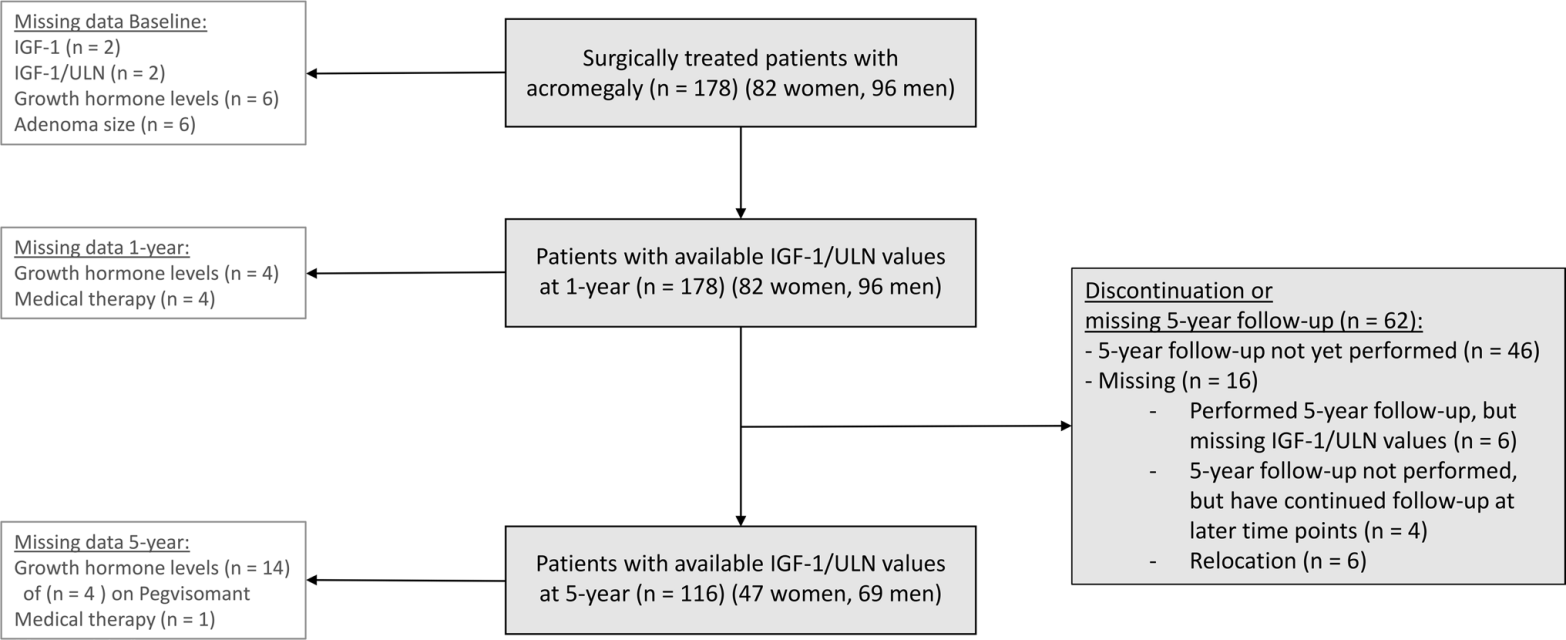
Flow diagram of included patients. Flow diagram of patients included in the study at one-year (*n* = 178) and five-year (*n* = 116) follow-up (grey boxes). Missing values at baseline, one-year and five-year follow-up are described in white boxes

A total of 178 patients diagnosed with acromegaly, were treated surgically and included in our database during the study period. Median age at diagnosis was 49.1 (37.6–58.7) years, and 46% were women. All patients had available IGF-1/ULN values at one-year, and 116 patients (65%) at five-year follow-up (Fig. 1).

Radiological assessments at baseline were available for 172 patients, of whom 24% had microadenomas (< 10 mm) and 76% had macroadenomas (≥ 10 mm). As first treatment for acromegaly, 48% received surgery, and 46% received 1^st^ generation SSAs. (Remaining baseline data are presented in Table 1).

**Table 1:** Baseline data in 178 patients with acromegaly

Variables	
Age at inclusion	49.1 (37.6–58.7)
Sex (women)	82 (46.1%)
IGF-1 (nmol/L)^a^	93.0 (64.4–125.0)
IGF-1/ULN^a^	2.6 (1.8–3.5)
Growth hormone (µg/L)^b^	6.9 (3.3–16.5)
Adenoma size (mm)^b^	14 (10–19)
Macroadenoma	131 (76.2%)
First treatment	
Surgery	83 (48.3%)
1^st^ generation SSAs	80 (46.5%)
Other b	9 (5.2%)

Baseline patient characteristics. Data are given in median (interquartile range) for continuous measures and total (%, percent) for categorical measures. ^a^*N* = 176. ^b^*N* = 172 and other includes dopamine agonists, growth hormone receptor antagonists and medical combination treatment. Abbreviations: Insulin-like growth factor 1 (IGF-1), upper limit of normal (ULN), somatostatin analogues (SSAs)

### Follow-up

Table 2 describes patient characteristics at one-year and five-year postoperative follow-up. Of 174 patients with available data about medical therapy, 67 (38%) received medical treatment for acromegaly at one-year follow-up. At five-year follow-up, 55 patients (48%) received medical treatment for acromegaly. In paired samples, there was no significant difference in the proportion of patients treated with medications for acromegaly at one-year compared to five-year follow-up (45% vs. 48%, *p* = 0.577). Of the 60 patients who did not receive any medical treatment for acromegaly at five-year, the median IGF-1/ULN was 0.8 (0.6–0.9, range: 0.2–1.3) and median GH was 0.5 (0.2–1.3) µg/L.

**Table 2:** One-year and five-year postoperative follow-up

Variables	One-year PO *N* = 178	Five-year PO *N* = 116^c^
IGF-1 (nmol/L)	28.1 (21.0–37.2)	23.8 (18.1–31.4)
IGF-1/ULN	0.9 (0.7–1.2)	0.8 (0.7–1.1)
Growth hormone (µg/L)	1.0 (0.4–2.6)^a^	0.6 (0.2–2.2)^d^
Medical therapy	^a^	^e^
None	107 (61.5%)	60 (52.2%)
1st generation SSAs	54 (31.0%)	37 (32.2%)
Pasierotide	3 (1.7%)	2 (1.7%)
DAs	3 (1.7%)	3 (2.6%)
GHRAs	1 (0.6%)	5 (4.3%)
Medical combination treatment	6 (3.5%)	8 (7.0%)
Radiotherapy^b^	0 (0%)	13 (11.2%)
Reoperated^b^	1 (0.6%)	14 (12.1%)

Patient characteristics at one-year and five-year postoperative follow-up. Data are given in median (interquartile range) for continuous measures and total (%, percent) for categorical measures. ^a^*N* = 174. ^b^Patients that received reoperation and/or radiation therapy within the follow-up. Radiotherapy was fractionated in six patients; of these one was treated with protons and five with photons. Seven patients were treated with stereotactic radiosurgery. ^c^Patients lacking five-year postoperative follow-up due to short follow-up time or missing, were censored (*N* = 62). ^d^*N* = 102. ^e^*N* = 115. Abbreviations: Insulin-like growth factor 1 (IGF-1), upper limit of normal (ULN), somatostatin analogues (SSAs), dopamine agonists (DAs), growth hormone receptor antagonists (GHRAs)

### Short-term and long-term biochemical control

At one-year postoperative follow-up (short-term), 93 patients (53%) achieved biochemical control after surgery without any additional treatment. Of the patients receiving additional treatment (medical therapy, radiation therapy and/or reoperation), an additional 43 patients (25%) achieved biochemical control. Overall, 136 patients (78%) were in biochemical control by combined treatment modalities (Table 3A). Of the 116 patients with available five-year follow-up data (long-term), 48 patients (41%) achieved biochemical control after surgery without any additional treatment. Biochemical control was achieved in another 47 patients (41%) receiving additional treatment for acromegaly, resulting in 95 patients (82%) in biochemical control in total (Table 3B). There was no significant change in the overall biochemical control in patients without additional treatment between one-year and five-year follow-up (*p* = 0.213; paired samples). Of the 14 patients who were reoperated, five (36%) were in biochemical control and without medical therapy. Of the 13 patients who received radiotherapy, two (15%) were in biochemical control and without medical therapy at the five-year follow-up. Biochemical control according to IGF-1/ULN ≤ 1.2 and random GH levels < 1 µg/L combined, are described in Supplementary File 1. According to the combined criteria, 53 patients (31%, *n* = 170) were in control at one-year, and 28 patients (27%, *n* = 102) at five-year follow-up.

**Table 3:** Biochemical control after surgery and adjunctive treatment at one-year and five-year follow-up

A. One-year follow-up	Surgery^a^	Medication^b^	Reintervention^c^	Total
Control	93 (53%)	42 (24%)	1 (1%)	136 (78%)
Not control	13 (8%)	25 (14%)	0 (0%)	38 (22%)
Total	106 (61%)	67 (38%)	1 (1%)	174 (100%)
B. Five-year follow-up				
Control	48 (41%)	39 (34%)	15 (13%)	95 (82%)
Not control	5 (4%)	16 (14%)	4 (3%)	21 (18%)
Total	53 (46%)^d^	55 (47%)^d^	19 (16%)	116 (100%)

Biochemical control in patients at one-year (A) and five-year (B) follow-up, defined as IGF-1/ULN ≤ 1.2. Values are given in number of patients and percent. ^a^Surgery includes patients operated once, who did not receive any additional treatment for acromegaly. ^b^Medication includes patients that have received medical treatment after first surgery. ^c^Reintervention includes patients that underwent reoperation and/or radiotherapy after first surgery. There is an overlap between the medication group and reintervention group as some patients received both. ^d^Percentages differs due to rounding

Biochemical control was achieved in 62% of microadenomas and 51% of macroadenomas at one-year follow-up, with no significant difference in control rate between micro- and macroadenomas (Table 4A, p = 0.271). Five years postoperatively, biochemical control rates were 58% for microadenomas and 37% for macroadenomas, with a trend towards higher control rate in microadenomas (Table 4B, p = 0.058). When investigating whether control rates for micro- and macroadenomas at the five-year follow-up changed when control was defined as IGF-1 < 1 × ULN, five patients with microadenomas changed status from controlled to not controlled, all of whom had low GH-levels (< 1 µg/L; median 0.2; IQR: 0.18–0.32).

**Table 4:** Biochemical control at one-year and five-year follow-up in micro- and macroadenomas

A. One-year follow-up	Control after first surgery^a^	Adjunctive treatment and/or not control^b^	Total^c^
Microadenoma	24 (62%)	15 (38%)	39 (100%)
Macroadenoma	66 (51%)	64 (49%)	130 (100%)
Total	90 (53%)	79 (47%)	169 (100%)
B. Five-year follow-up			
Microadenoma	14 (58%)	10 (42%)	24 (100%)
Macroadenoma	32 (37%)	55 (63%)	87 (100%)
Total	46 (41%)	65 (59%)	111 (100%)

Biochemical control defined as IGF-1/ULN ≤ 1.2 in micro- and macroadenomas at one-year and five-year follow-up. Values are given in number of patients and percent. ^a^Biochemical control after first surgery without any additional treatment for acromegaly. ^b^Patients receiving adjunctive treatment after first surgery, with or without biochemical control, and patients not in biochemical control without additional treatment. ^c^Missing patients are presented in Fig. 1

### Factors predicting biochemical control

At short-term the only factor significantly predicting biochemical control was the absolute value for IGF-1 (nmol/L) at baseline (OR: 0.97 (95% CI 0.94–0.99), *p* = 0.045). Neither age, sex, GH, IGF-1 (nmol/L) and IGF-1/ULN levels, tumor size (macroadenoma vs. microadenoma) and first treatment modality (surgery vs. 1^st^ generation SSAs) predicted control at long-term follow-up.

## Discussion

In the present, single-center cohort study of prospectively enrolled patients with newly diagnosed acromegaly from South-Eastern Norway, we found that the rates for biochemical control achieved by the first surgery were 53% at short-term and 41% at long-term follow-up. However, with adjunctive treatment after first surgery, the majority of patients obtained control long-term. We found a trend towards higher control rates in microadenomas as compared to macroadenomas at long-term.

The results of overall biochemical control rates in the current study are in line with others, despite differences in biochemical control criteria, duration of follow-up and study design [4, 17, 21, 29]. We found a distribution of micro- and macroadenomas in our cohort similar to other studies [4, 21, 27]. However, the control rates for microadenomas in our results were lower than previously reported by some [17, 28, 29], but similar to the real-life data by Mercado et al. [21]. Lower biochemical control rates are reported in tumors with cavernous sinus invasion [17, 21], but data on cavernous sinus invasion were not analyzed in our cohort. Earlier diagnosis of acromegaly could potentially prevent parasellar extension/cavernous sinus invasion. Thus, a decrease in diagnostic delay is of importance to improve surgical cure. We demonstrated that with adjunctive treatment the proportion of patients in biochemical control increased by two-fold at long-term follow-up. In comparison, a register-based study from Belgium described an increase in patients achieving biochemical control, from 34 to 42%, when receiving adjunctive medical treatment and/or radiotherapy [4]. Furthermore, in a Chinese single-center study of 659 patients with acromegaly, patients in biochemical control increased from 55 to 69% with additional treatment at long-term [17]. Thus, the intensified and multimodal treatment improves biochemical control, and is probably an important factor contributing to the normalized mortality in acromegaly in recent studies [3, 12].

Comparing surgical cure rates between different centers has proved challenging due to the large variety in biochemical control criteria and definition of outcomes [1, 35]. The criteria for biochemical control have constantly been evolving. Analytical, physiological, pathological and pharmacological factors affect the GH and IGF-1 levels, and can result in discrepancies in measurements [25]. Thus, strict cut-off criteria in defining biochemical control do not necessarily reflect the true clinical picture [16]. Further, gonadal status and body mass index should be taken into consideration, when assessing GH levels [26]. Biochemical control according to IGF-1/ULN ≤ 1.2 and random GH levels < 1 µg/L combined, showed lower control rates in our results. Low random GH-levels can be used to determine control, but high random GH-levels do not necessarily reflect lack of biochemical control. The oral glucose tolerance test (OGTT) is necessary to determine if GH-nadir levels are elevated, but these were routinely not measured at the one-year and five-year follow-up in our cohort. In the present study, we demonstrated that few patients changed from being in control to not control, when changing the remission criterion cut-off to IGF-1/ULN < 1. Yet, according to the random morning GH-levels for all these patients, they were indeed in biochemical control. Therefore, we found that IGF-1/ULN levels with a slightly higher cut-off for biochemical control was appropriate, and also in line with a recent consensus statement [16].

We found that lower absolute IGF-1 levels at diagnosis were associated with biochemical control after surgery at short-term in corroboration with other studies [11, 30, 31], but not with IGF-1/ULN and not at long-term follow-up. Thus, we do not regard this finding as a helpful predictor of biochemical control. Moreover, we did not find any variables associated with biochemical control at long-term. Of the few studies reporting long-term follow-up data exceeding three years postoperatively, preoperative GH and IGF-1 levels, tumor size and invasiveness, and age at diagnosis were among factors predicting long-term biochemical control after surgery [11, 17, 30, 31, 33]. Presurgical medical treatment may improve control rates [15], but medical pretreatment as a systematic approach in unselected patients remains controversial [8, 9, 15, 22, 34]. A multidisciplinary, individualized approach is recommended for the management of acromegaly and may also improve surgical outcomes [1, 6, 14, 16, 18, 20].

In the most recent guidelines, surgery is recommended as primary treatment for most patients with acromegaly, and to maximize surgical cure, experienced pituitary surgeons are recommended to perform the procedure [18]. Recent large series have reported surgical cure rates in less than half of the patients, comparable to our results [4, 17, 21]. Studies have so far not demonstrated clear differences in surgical cure rates between endoscopic and microscopic approaches [2, 10, 28, 35]. In recent data, the endoscopic approach has been described as a predictor of long-term biochemical control [17]. We converted from microsurgical to endoscopic transsphenoidal surgery in 2006. The learning curve was steep, and the transition may have influenced our findings during the early period of the study. In 2019 we adopted the “four hand technique” where the assistant handles the endoscope and provides visualization, whereas the surgeon performs two hands microsurgery through both nostrils [32]. In an ongoing prospective study we do investigate whether the resection rate and surgical cure rate will be improved by this method. Although the overall surgical cure rates remain modest, mortality in patients with acromegaly has decreased over the last two decades, approximating mortality rates of the general population [5], reflecting the development of new treatment approaches and modalities. We have recently confirmed this for our population cohort in South-Eastern Norway, where the mortality in patients with acromegaly was not increased compared to the general population [12]. Nevertheless, it remains of importance to improve surgical outcomes, and hereby reduce morbidity and complicated, costly and long-term treatment, for both patients and the society.

The strengths of this study are the register-based design with prospectively collected data and long-term follow-up of unselected patients, representative for the population in South-Eastern Norway. However, detailed surgical, radiological and immunohistochemical data, which could provide further information on potential factors and tumor characteristics predicting biochemical control, were not available for this study. Potential predictors of biochemical control only assessed by tumor size, but not Knosp based cavernous sinus invasion, which is more specific for pituitary lesions, is a limitation. There is also a risk of Type 2 error, especially for the five-year data, with fewer observations in the analyses of factors predicting biochemical control long-term.

## Conclusion

In the present series of patients treated with pituitary surgery for GH-producing adenomas, we demonstrated similar rates of biochemical control compared to other recent series. Efforts are needed to decrease the diagnostic delay and improve therapeutic methods to increase surgical cure rates.

### Supplementary Information

Below is the link to the electronic supplementary material.

Supplementary file1 (XLSX 10 KB)

## Data Availability

The data supporting the findings of this study are not openly available due to restrictions as they contain information that could compromise the privacy of research participants and due to restrictions in the institutions data protection policy. The data are available upon reasonable request from the corresponding author.
